# Supplementary material for: Elucidating the correlations between cancer initiation times and lifetime cancer risks
Source: Sci Rep. 2019 Dec 12;9:18940. doi: 10.1038/s41598-019-55300-w (PMC6908632; doi:10.1038/s41598-019-55300-w)
Supplement: Supplementary file 1 — Supporting Information [file 41598_2019_55300_MOESM1_ESM.pdf]

Elucidating the correlations between cancer initiation times and lifetime cancer risks

Hamid Teimouri, Maria Kochugaeva and Anatoly B Kolomeisky

In this supporting information we provide details of calculations for the equations in the main text.

1. Calculating transition rates a_n

To compute the transition rate between the states $(n, N-n)$ (n mutated cells and $N-n$ normal cells) and $(n+1, N-n-1)$ ($n+1$ mutated cells and $N-n-1$ normal cells), we consider the cell replication as a two-state process, as shown in Fig. S1. First, the randomly chosen cell is divided and the number of cells in the tissue increases to $N+1$. Then immediately one of the randomly chosen cells is removed to keep the total number of cells equal to N. From the state $(n, N-n)$ our system goes to an intermediate state $(n+1, N-n)$. This corresponds to the division by the mutated cell. The rate for this process is equal to rbn because there are n mutated cells, each of them can divide with the rate rb. The reverse transition from the intermediate state $(n+1, N-n)$ with $N+1$ total number of cells to the state $(n, N-n)$ with N total cells is equal to $A(n+1)$. Here A is the rate of removal of any randomly chosen cell from the system (here we assume that $A \gg b$). From the intermediate state $(n+1, N-n)$ the system can also go the state $(n+1, N-n-1)$ with the rate $A(N-n)$. One can easily evaluate then the effective time to go from the state $(n, N-n)$ to the state $(n+1, N-n-1)$ using a first-passage method [1]. For simplicity, we rename the states with labels 0, 1, and 2 as shown in Fig. S1. Then we define $\Pi_i(t)$ as the probability for the system to reach the state 2 if at $t=0$ the system was in the state i ($i = 0$, 1, or 2). These probabilities are governed by the backward master equations [1]:

$$\frac{d\Pi_0(t)}{dt} = rbn\Pi_1(t) - rbn\Pi_0(t); \tag{S1}$$

$$\frac{d\Pi_1(t)}{dt} = A(n+1)\Pi_0(t) + A(N-n)\Pi_2(t) - (A(n+1) + A(N-n))\Pi_1(t). \tag{S2}$$

In addition, we have the following boundary condition $\Pi_2(t) = \delta(t)$. These equations can be solved by using Laplace transformations, yielding

$$(s + rbn)\widetilde{\Pi_0}(s) = rbn\widetilde{\Pi_1}(s) \tag{S3}$$

$$(s + A(N-n) + A(n+1))\widetilde{\Pi_1}(s) = A(N-n) + A(n+1)\widetilde{\Pi_0}(s) \tag{S4}$$

From these equations we obtain,

$$\widetilde{\Pi_0}(s) = \frac{rbnA(N-n)}{s^2 + s(rbn + A(N-n) + A(n+1)) + rbnA(N-n)} \quad \text{(S5)}$$

The mean first-passage time $T_{n,n+1}$ to reach the state $(n+1, N-n-1)$ from the state $(n, N-n)$ is given by:

$$T_{n,n+1} = \frac{-\frac{\partial \widetilde{\Pi_0}}{\partial s}|_{s=0}}{\widetilde{\Pi_0}(s=0)} \quad \text{(S6)}$$

After some algebra, the expression for the mean first-passage time can be written as

$$T_{n,n+1} = \frac{rbn + A(N-n) + A(n+1)}{rbnA(N-n)} = \frac{1}{rbn} + \frac{1}{A(N-n)} + \frac{A(n+1)}{rbnA(N-n)}, \quad \text{(S7)}$$

which is equal to the inverse transition rate between these states. This equation can be explained in the following way. The first two terms correspond to transitions where the system goes directly from the state $(n, N-1)$ to the state $(n+1, N-n-1)$ in two sequential steps without backward transitions from the intermediate state, while the third term corresponds to all other trajectories that include the backward steps. From this expression, we derive (recalling that $A \gg r$),

$$ra_n = \frac{rbnA(N-n)}{rbn + A(N+1)} \simeq \frac{rbn(N-n)}{N+1}. \quad \text{(S8)}$$

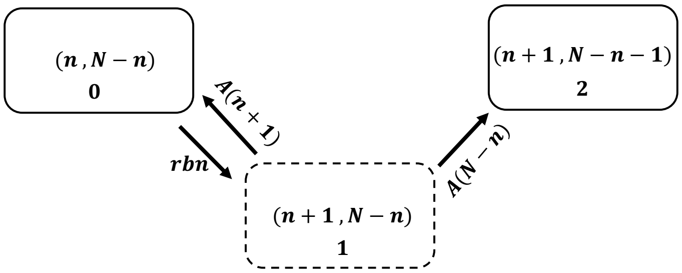

Figure S1. Schematic view for the derivation of Eq. S8 .

2. Calculation of fixation probability

Consider a tissue compartment that has N normal cells. At time zero one of them is mutated. Normal cells divide with a speed b, while the mutated cell divides with a rate r (in units of b). Assuming that the compartment always has the same number of cells, let us investigate the dynamics of how the whole compartment can become full of mutant cells.

The problem is analogous then to a random walk on the lattice of N sites. At $t = 0$ the walk starts at the site 1. The state n corresponds to n mutated and $(N-n)$ normal cells. As shown above, the transition rate from the state n to $n+1$ is equal to ra_n, where

$$a_n = b\frac{n(N-n)}{N+1} \quad \text{(S9)}$$

The backward transition (from the state n to the state $n-1$) is equal to a_n. The problem of understanding when the whole compartment becomes mutated is analogous then to a first-passage problem of the random walker starting on the site 1 to reach the site N for the first time before disappearing to the site 0. One can define the corresponding first-passage probability density functions to start from any site n and reach the site N at time t (if at $t = 0$ the k was at the site n), $F_n(t)$. The temporal evolution of these probabilities follows the backward master equations

$$\frac{dF_n(t)}{dt} = ra_nF_{n+1}(t) + a_nF_{n-1}(t) - a_n(1+r)F_n(t), \tag{S10}$$

for $1 < n < N$; and

$$\frac{dF_1(t)}{dt} = ra_1F_2(t) - a_1(1+r)F_1(t). \tag{S11}$$

In addition, we have the boundary condition $F_N(t) = \delta(t)$, which means that the process is immediately accomplished if the walker starts from the site N. Let us also do the calculations assuming $b = 1$, i.e., all times scales are renormalized with respect to cell replication time.

It is convenient to solve this problem using the Laplace transformation, which changes the backward master equations:

$$\frac{s\widetilde{F_n}}{ra_n} + (1+1/r)\widetilde{F_n} = \widetilde{F_{n+1}} + \frac{1}{r}\widetilde{F_n} \tag{S12}$$

$$\frac{s\widetilde{F_1}}{ra_1} + (1+1/r)\widetilde{F_1} = \widetilde{F_2} \tag{S13}$$

and $F_N = 1$. Because we are interested only in the fixation probabilities and fixation times, there is no need to obtain full analytical expressions for F_n, but it is needed to determine the expansion of this function up to the linear term in s. Thus, we can write

$$\widetilde{F_n}(s) \simeq \pi_n + b_n\frac{s}{r} \tag{S14}$$

where $\pi_n = \widetilde{F_n}(s=0)$, is the fixation probability starting from n single mutations, and the unknown parameters b_n are related to the fixation times (viewed as conditional mean first-passage times) as

$$T_n = \frac{-\frac{\partial\widetilde{F_n}}{\partial s}|_{s=0}}{\widetilde{F_n}(s=0)} = \frac{b_n}{f_n} \tag{S15}$$

Note that $\pi_N = 1$ and $b_N = 0$. Substituting Eq. S14 into Eqs. S12 and S13 we obtain for the fixation probabilities

$$(1+1/r)\pi_n = \pi_{n+1} + \left(\frac{1}{r}\right)\pi_{n-1} \tag{S16}$$

$$(1+1/r)\pi_1 = \pi_2 \tag{S17}$$

These equations can be easily solved, leading to the following explicit expressions for the fixation probability (a well-known result),

$$\pi_n = \frac{1-1/r^n}{1-1/r^N}. \tag{S18}$$

3. Calculation of fixation times

From Eqs. S12, S13 and S14, the corresponding equations for parameters b_n can be written as,

$$\frac{\pi_n}{a_n} + (1 + \frac{1}{r}) b_n = b_{n+1} + \frac{b_{n-1}}{r}, \tag{S19}$$

$$\frac{\pi_1}{a_1} + (1 + \frac{1}{r}) b_1 = b_2. \tag{S20}$$

To solve Eqs. S19 and S20, let us write the following anzats

$$b_{n+1} = b_n + K_n, \tag{S21}$$

where K_n is another unknown parameter that will be determined. Then the substitution of Eq. S21 into Eqs. S19 and S20 yields

$$K_n - \frac{K_{n-1}}{r} = \frac{\pi_n}{a_n}; \tag{S22}$$

$$K_1 = \frac{\pi_1}{a_1} + \frac{b_1}{r}. \tag{S23}$$

Eq. S22 can be easily solved, producing

$$K_n = \sum_{l=0}^{n-2} \frac{1}{r^l} \frac{\pi_{n-l}}{a_{n-l}} + \frac{K_1}{r^{n-l}}. \tag{S24}$$

Then from Eq. S21 we can write

$$b_n = b_1 + \sum_{j=1}^{n-1} K_j = b_1 (1 + \frac{1}{r} + ... + \frac{1}{r^{n-1}}) + \sum_{j=1}^{n-1} \sum_{l=0}^{j-1} \frac{1}{r^l} \frac{\pi_{j-l}}{a_{j-l}}. \tag{S25}$$

This expression is valid for any $1 \le n \le N$, which due to $b_N = 0$ leads to

$$b_1 = -\frac{\sum_{j=1}^{N-1} \sum_{l=0}^{j-1} (\frac{\pi_{j-l}}{r^l a_{j-l}})}{1 + \frac{1}{r} + ... + \frac{1}{r^{N-1}}} \tag{S26}$$

Then the final fixation time (normalized to the cell replication rate b) will be equal

$$T_1 = -\frac{b_1}{r\pi_1} = \frac{\sum_{j=1}^{N-1} \sum_{l=0}^{j-1} \frac{1}{r^l} \frac{\pi_{j-l}}{a_{j-l}}}{r\pi_1 (1 + \frac{1}{r} + ... + \frac{1}{r^{N-1}})}, \tag{S27}$$

from which after some algebra we obtain

$$T_1 = \frac{N+1}{r(1 - r^{-N})} \sum_{j=1}^{N-1} \sum_{l=0}^{j-1} \frac{(r^{-l} - r^{-j})}{(j-l)(N-j+l)}. \tag{S28}$$

The summations can be further simplified by defining a new index $n = j - l$.

$$\sum_{j=1}^{N-1} \sum_{l=0}^{j-1} \frac{(r^{-l} - r^{-j})}{(j-l)(N-j+l)} = \sum_{j=1}^{N-1} \sum_{l=0}^{j-1} \frac{(r^n - 1)}{n(N-n)} \frac{1}{r^j}. \tag{S29}$$

Now we can change the order of summations,

$$\sum_{j=1}^{N-1}\sum_{l=0}^{j-1}\frac{(r^n-1)}{n(N-n)}\frac{1}{r^j}=\sum_{n=1}^{N-1}\frac{(r^n-1)}{n(N-n)}\sum_{j=n}^{N-1}\frac{1}{r^j}. \tag{S30}$$

This eventually produces a compact expression,

$$T_1=\frac{N+1}{b}\sum_{n=1}^{N-1}\frac{1}{n(N-n)}\left(\frac{r^n-1}{r-1}\right)\left(\frac{r^{N-n}-1}{r^N-1}\right). \tag{S31}$$

It can be further simplified for $r\to 1$ by employing L'Hôpital's rule:

$$T_1=\frac{N+1}{b}\frac{N-1}{N}, \tag{S32}$$

which in the limit $N\to\infty$ gives

$$T_1\simeq\frac{N}{b}. \tag{S33}$$

4. Explicit expression for fixation times for $N\to\infty$

In general it is difficult to perform explicit summation in Eq. S27 for large N. For $N\to\infty$, we can convert summation to integration:

$$\sum_{j=1}^{N-1}\sum_{l=0}^{j-1}\frac{(r^{-l}-r^{-j})}{(j-l)(N-j+l)}\simeq\int_1^{N-1}dy\int_0^{y-1}\frac{dx(e^{cx}-e^{cy})}{(y-x)(N-y+x)}, \tag{S34}$$

where $c=-\ln r$. This integral can be written as:

$$\begin{aligned}&\int_1^{N-1}dy\int_0^{y-1}\frac{dx(e^{cx}-e^{cy})}{(y-x)(N-y+x)}=\frac{1}{N}\int_1^{N-1}dy\int_0^{y-1}dx(e^{cx}-e^{cy})\left(\frac{1}{y-x}+\frac{1}{N-y+x}\right)\\ &=\frac{1}{N}\left[\int_1^{N-1}dy\int_0^{y-1}\frac{e^{cx}}{y-x}dx+\int_1^{N-1}dy\int_0^{y-1}\frac{e^{cx}}{N-y+x}dx-\int_1^{N-1}dye^{cy}\int_0^{y-1}\frac{dx}{y-x}-\int_1^{N-1}dye^{cy}\int_0^{y-1}\frac{dx}{N-y+x}\right].\end{aligned} \tag{S35}$$

Now we perform integrals term by term:

$$\int_1^{N-1}dy\int_0^{y-1}\frac{e^{cx}}{y-x}dx=-\frac{\mathrm{Ei(c)}}{c}[e^{c(N-1)}-e^c]-\frac{1}{c}\left[\gamma+\ln(-cN)-e^{cN}\mathrm{Ei(-cN)}\right] \tag{S36}$$

$$\int_1^{N-1}dy\int_0^{y-1}\frac{e^{cx}}{N-y+x}dx=\frac{\mathrm{Ei(c(N-1))}}{c}[e^{-c}-e^{-c(N-1)}]-\frac{1}{c}\left[\gamma+\ln(-cN)-e^{cN}\mathrm{Ei(-cN)}\right] \tag{S37}$$

$$\int_1^{N-1}dye^{cy}\int_0^{y-1}\frac{dx}{y-x}=\frac{1}{c}[-\mathrm{Ei(c(N-1))}+\mathrm{Ei(c)}+\mathrm{e^{c(N-1)}}\ln(\mathrm{N}-1)] \tag{S38}$$

$$\int_1^{N-1}dye^{cy}\int_0^{y-1}\frac{dx}{N-y+x}=\frac{e^{cN}}{c}[-\mathrm{Ei(-c(N-1))}+\mathrm{Ei(-c)}+\mathrm{e^{-c}}\ln(\mathrm{N}-1)]. \tag{S39}$$

After some algebra, we obtain:

$$\begin{aligned}&\int_1^{N-1}dx\int_0^{y-1}dy(e^{cx}-e^{cy})\left(\frac{1}{y-x}+\frac{1}{N-y+x}\right)=\\ &e^{cN}\left[-\frac{\mathrm{Ei(c)}}{c}e^{-c}+2\frac{\mathrm{Ei(-cN)}}{c}+\frac{\mathrm{Ei(-c(N-1))}}{c}-\frac{\mathrm{Ei(-c)}}{c}-2e^{-c}\frac{\ln(N-1)}{c}\right]\\ &+\mathrm{Ei(c(N-1))}\left[\frac{\mathrm{e^{-c}}}{\mathrm{c}}-\mathrm{e^{-c(N-1)}}+\frac{1}{\mathrm{c}}\right]+\frac{\mathrm{Ei(c)}}{\mathrm{c}}\mathrm{e^c}-\frac{2}{\mathrm{c}}(\gamma+\ln(-\mathrm{cN}))-\frac{\mathrm{Ei(c)}}{\mathrm{c}},\end{aligned} \tag{S40}$$

where Ei(x) represents exponential function defined by $\mathrm{Ei(x)} = -\int_{-x}^{\infty} \frac{e^{-z}}{z} dz$, and γ is the Euler–Mascheroni constant. Therefore the fixation time is given by

$$T_1 = \frac{e^{cN}}{r(1-r^{-N})}\left[-\frac{\mathrm{Ei(c)}}{c}e^{-c} + 2\frac{\mathrm{Ei(-cN)}}{c} + \frac{\mathrm{Ei(-c(N-1))}}{c} - \frac{\mathrm{Ei(-c)}}{c} - 2e^{-c}\frac{\ln(N-1)}{c}\right]$$
$$+\frac{\mathrm{Ei(c(N-1))}}{r(1-r^{-N})}\left[\frac{e^{-c}}{c} - e^{-c(N-1)} + \frac{1}{c}\right] + \frac{1}{r(1-r^{-N})}\left[\frac{\mathrm{Ei(c)}}{c}e^{c} - \frac{2}{c}(\gamma + \ln(-cN)) - \frac{\mathrm{Ei(c)}}{c}\right]. \quad \text{(S41)}$$

Because $c < 0$ and $N \to \infty$, then the first two terms vanish and thus we finally obtain:

$$T_1 \simeq \frac{1}{r(1-r^{-N})}\left[-\frac{\mathrm{Ei(-\ln r)}}{r\ln r} + \frac{2}{\ln r}(\gamma + \ln(N\ln r)) + \frac{\mathrm{Ei(-\ln r)}}{\ln r}\right] \quad \text{(S42)}$$

Because the number of stem cells is very large, it can be shown from Eq. 12 in the main text and (S42) that

$$T_1 = \frac{1}{r(1-r^{-N})}\left[\mathrm{Ei}\left(-\frac{\mathrm{R_{ltr}}}{\mathrm{bT_{life}}\mu\mathrm{Q_{pr}N}}\right)\right]\frac{1}{b} + \frac{2\mu Q_{pr}N}{r(1-r^{-N})R_{ltr}}\left[\gamma + \ln\left(\frac{R_{ltr}}{bT_{\text{life}}\mu Q_{pr}}\right)\right]T_{\text{life}} \quad \text{(S43)}$$

5. Varying probabilities of cancer progression and oncogene activation

Since our theoretical predictions depend strongly on the probability of cancer progression (Q_{pr}) and the probability of the appearance of mutation (μ), which are not well determined in the literature, we varied these parameters as shown in Fig. S2. One can see that the calculated fixation times are sensitive to variations in these parameters.

In addition, Fig. S3 presents the correlation analysis between cancer initiation times and cancer lifetime risks for two other sets of the parameters μ and Q_{pr}. In both cases, no correlations are found.

6. References

[1] Anatoly B Kolomeisky. *Motor proteins and molecular motors*. CRC Press, 2015.

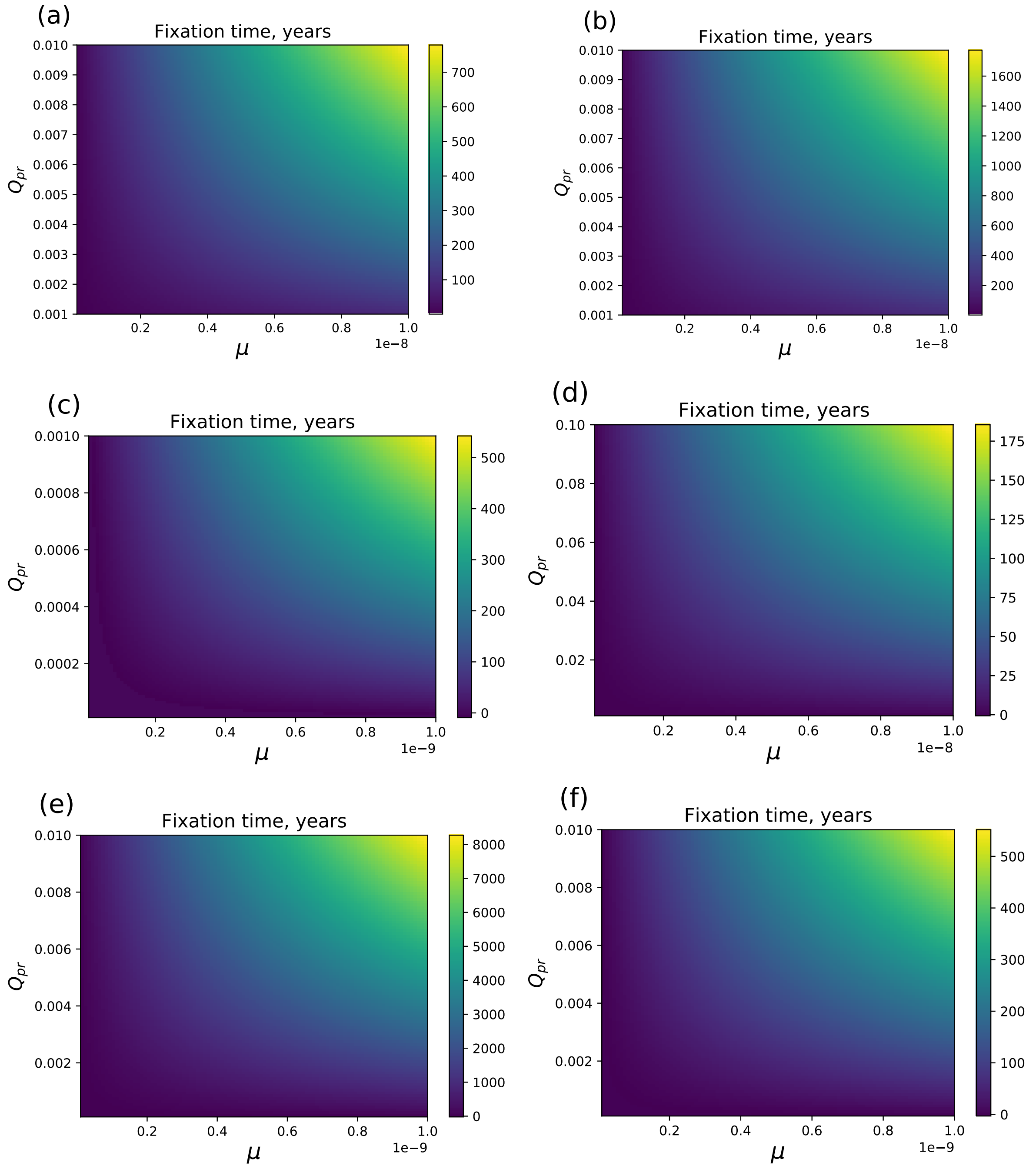

Figure S2. Extinction time T_1 over $\mu - Q_{pr}$ parameter space for (a) Colorectal adenocarcinoma (b) Small intestine adenocarcinoma (c) Melanoma (d) Duodenum adenocarcinoma with FAP (e) Pancreatic ductal adenocarcinoma (f) Basal cell carcinoma.

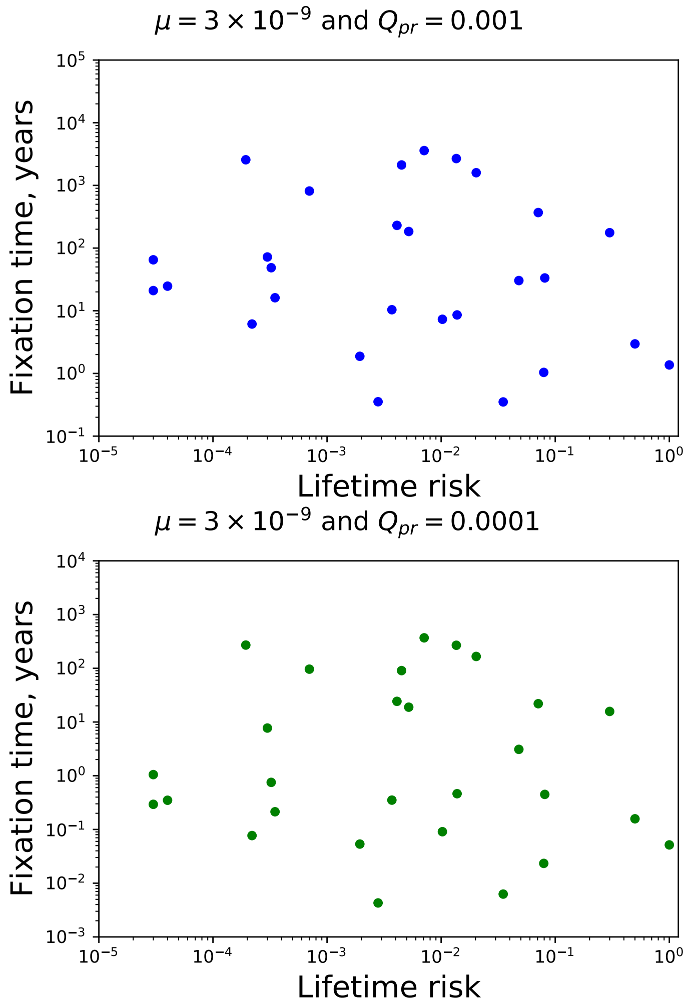

Figure S3. Fixation time vs lifetime risk for different types of cancer. Correlation analysis yields a Spearman's correlation coefficient of −0.14 and p-value of 0.46 for the top plot and Spearman's correlation coefficient of −0.08 and p-value of 0.69 for the bottom plot.
